# Near-Peer-Led Spontaneous Vaginal Delivery Simulation Improves Clerkship Students’ Knowledge and Confidence

**DOI:** 10.1177/23821205261459895

**Published:** 2026-06-08

**Authors:** Claire M. Schenken, Nisha Kalyanpur, Malavika Perinchery, Jacqueline L. Moreno, Nicholas Stansbury, Gabriel Medrano-Valle, Sarah M. Page-Ramsey, Erin L. Nelson

**Affiliations:** 1 Department of Obstetrics and Gynecology, 14742 University of Texas Health Science Center San Antonio, San Antonio, TX, USA; 2 Department of Obstetrics and Gynecology, 12334 University of Texas Southwestern, Dallas, TX, USA; 3 Department of Obstetrics and Gynecology, 2462 University of Chicago, Chicago, IL, USA

**Keywords:** simulation, obstetrics, medical education

## Abstract

**Introduction:**

This study aims to evaluate a near-peer-led, low-fidelity spontaneous vaginal delivery (SVD) simulation on Obstetrics and Gynecology (OB/Gyn) clerkship medical students’ knowledge, self-perceived confidence, and experiences.

**Methods:**

A pre-post intervention study was performed. Senior undergraduate medical students (MS4s) served as near-peer teachers and presented an SVD lecture and simulation session using a low-fidelity manikin to clerkship students on the first day of the Ob/Gyn clerkship. Demographics, knowledge, confidence, and experience were assessed via anonymous surveys at three points: pre-simulation, post-simulation, and post-clerkship. Students who completed the rotation in the preceding 1 year (prior to simulation implementation) served as controls.

**Results:**

259 clerkship students completed pre- and post-simulation surveys with 71 also completing post-clerkship surveys. 54 students served as controls. Post-simulation knowledge scores were significantly greater than pre-simulation and controls (76.9% vs. 54.5%, p< 0.01; 76.9% vs. 53.3%, p < .01). Knowledge increased, though not significantly, from post-simulation to post-clerkship (76.9% vs. 84.1%, p=0.07). Ob/Gyn clerkship student confidence significantly increased for all components of the vaginal delivery immediately following simulation (p <.01). Following the clerkship, students were more confident delivering and examining the placenta than immediately following the simulation (3.6 vs. 3.4, p=0.03; 3.6 vs. 3.3, p=0.04, respectively). Participation in delivery of placentas and infants did not significantly differ between students who completed the simulation and controls (3(3) vs. 3(3), p=0.32; 1(2) vs. 2(4), p=0.32, respectively).

**Conclusion:**

A 90-minute SVD simulation session at the start of the Ob/Gyn clerkship led by near-peers is feasible and significantly increases clerkship students’ knowledge and confidence to participate in an SVD.

## Introduction

The spontaneous vaginal delivery (SVD) is an important fundamental skill generally learned on the Obstetrics and Gynecology (Ob/Gyn) clerkship. However, numerous factors have contributed to a decreased volume of deliveries and other obstetric clinical opportunities for clerkship students. The role of simulation in undergraduate Ob/Gyn education has rapidly expanded to provide hands-on learning opportunities to students. Simulation training offers learners the opportunity to acquire Ob/Gyn knowledge, confidence, and skills in a controlled learning environment.

The literature suggests that SVD simulation increases medical students’ knowledge, self-perceived preparedness and confidence to perform a vaginal delivery.^1-5^ However, challenges to simulation during the Ob/Gyn clerkship include cost and distribution of learners and instructors across multiple clinical sites. Some of these barriers may be alleviated by senior undergraduate medical students serving as near-peer instructors of a simulation session given during regularly scheduled clerkship time. Benefits of near-peer teaching have been well-described in the literature and include non-inferior education, resource allocation, a calm and supportive learning environment, and cognitive congruence, which is the idea that a teacher who has similar prior knowledge and experiences to a learner may provide a more appropriate framework to help the learner understand.^6^ However, near-peer teaching in Ob/Gyn has not been well-investigated. To our knowledge, this is the first study that assesses the impact of a pilot lecture and simulation led by a senior undergraduate medical student for third-year medical student (MS3) Ob/Gyn clerkship students.

In our study, fourth-year senior undergraduate medical students (MS4) who successfully completed the Ob/Gyn clerkship were recruited to serve as near-peer simulation instructors. We hypothesized that a lecture and simulation taught by near-peers on a low-fidelity manikin would significantly increase clerkship students’ knowledge and confidence to perform a vaginal delivery.

## Materials and Methods

Clerkship students were divided into groups of three to six, according to their schedule. Ob/Gyn clerkship students were administered the lecture and simulation during regularly scheduled didactic time. Participants were included if they attended the lecture and simulation and completed the pre- and post-clerkship surveys. Clerkship students received a 30-minute lecture covering the following Association of Professors of Gynecology and Obstetrics (APGO) objectives: 1) Differentiate between the signs and symptoms of true labor and false labor; 2) Perform an initial assessment of a laboring patient; 3) Describe the four stages of labor and recognize common abnormalities; 4) Describe the steps of a vaginal delivery; and 5) List indications for operative delivery.^7^ Lecture slides were developed with two Ob/Gyn faculty members and one Ob/Gyn resident. Following the lecture, a simulated vaginal delivery was demonstrated by the instructor, and students had approximately 30 minutes to practice using the Gaumard Advanced Childbirth Simulator, a low-fidelity manikin. Finally, each student underwent a vaginal delivery simulation using the manikin and was provided immediate feedback from the instructor using a vaginal delivery procedural checklist previously described by Nitsche et al.^1^

Lectures and simulations were taught by MS4 near-peer instructors who had successfully completed the Ob/Gyn clerkship and expressed interest in teaching medical students. Students were recruited during group advising sessions for students applying to Ob/Gyn residency. Near-peer instructors underwent training including receiving the lecture and simulation by Ob/Gyn faculty and a senior resident prior to leading the educational sessions. Student instructors completed mock presentations and simulations with feedback. One near-peer instructor led each lecture and simulation study.

Anonymously linked surveys were sent to consenting participants at 3 separate time-points: pre-simulation, post-simulation (to be completed within 24 hours of simulation session), and post-clerkship (to be completed within 7 days of Ob/Gyn clerkship). The surveys comprised of 12, 7, and 11 items, respectively (see Appendix A). A 12-item survey was also distributed to all senior medical students who had completed the Ob/Gyn clerkship in the preceding 1 year and had not received simulation training during their clerkship (see Appendix A). These medical students served as a secondary control group. Surveys included demographic information, interest in Ob/Gyn, questions on experience observing and performing vaginal deliveries, knowledge about normal labor and delivery, and confidence to participate in and perform a vaginal delivery. Two co-authors developed the surveys using described APGO objectives and by adapting a previously published survey about medical student confidence to participate in a vaginal delivery.^8,9^ Surveys were piloted by 20 senior medical students, Ob/Gyn residents, and Ob/Gyn faculty at UT Health San Antonio prior to use in this course.

### Statistical Analysis

Demographics and experience are described using descriptive statistics and the Chi-square test. Experience data is reported as median (interquartile range (IQR)). Responses on the survey were compared between the 2021-2022 cohort (no simulation) and the 2022-2024 cohorts using Mann-Whitney *U* tests. Student responses to the surveys were compared pre- and post-simulation and post-clerkship using Wilcoxon signed-rank tests. Statistical analysis was performed using GraphPad Prism Version 10.3.1 (GraphPad Software, San Diego, CA). Significance was determined at the *p* < .05 level.

## Results

A total of 342 clerkship students received simulation training. 259 clerkship students (75.7%) received simulation training and completed both pre- and post-simulation surveys. 71 clerkship students (20.8%) completed pre-simulation, post-simulation, and post-clerkship surveys. 12 students did not complete any survey (3.5%). The control group was comprised of 54 students who completed the Ob/Gyn clerkship in the 1 year preceding the initiation of simulation sessions, for a response rate of 28.9%. Demographics, Ob/Gyn interest, and prior SVD exposure are described in Table 1.

**Table 1.** Demographics, Interest, and Prior Exposure for Simulation Students Who Completed all Surveys and Control Students

​		Simulation students (n=71)	Control students (n=54)	*P* Value
Gender	Male	32 (45.1%)	25 (46.3%)	0.94
	Female	38 (53.5%)	29 (53.7%)	
	Non-binary	1 (1.4%)	​	​
Semester	Fall	37 (52.1%)	23 (42.6%)	0.29
	Spring	34 (47.9%)	31 (57.4%)	
Ob/Gyn Interest	I do not plan on going into Ob/Gyn	44 (62.0%)	44 (81.5%)	<.01
	I am not sure	18 (25.4%)	3 (5.6%)	
	I plan to go into Ob/Gyn	9 (12.7%)	7 (13.0%)	
Prior SVD Exposure	None	42 (59.2%)	​	​
	Observed 1 SVD	14 (19.7%)	​	​
	Observed 2+ SVDs	13 (18.3%)	​	​
	Performed 1 vaginal delivery with assistance	2 (2.8%)	​	​

The proportion of males and females did not significantly differ between simulation and control students (p = 0.94). Clerkship semester did not significantly differ between simulation and control students (p=0.29). There was a significant difference between control and simulation groups and interest in going into Ob/Gyn (p < .01). This may be at least partially explained by the control group’s senior status, as those who have completed clerkships may be more likely to know which specialty they will apply to.

### Knowledge

**Figure 1.**
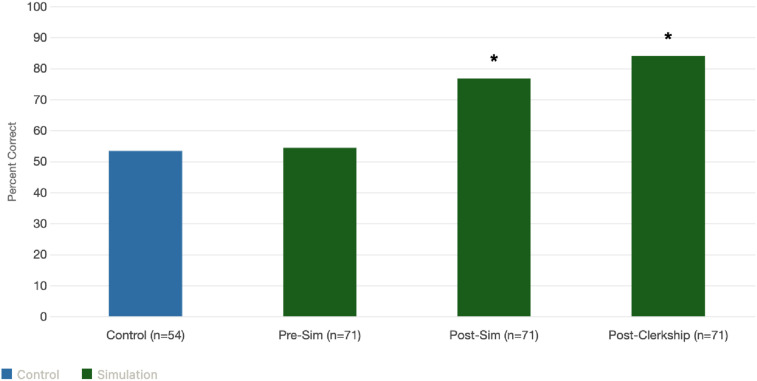
Knowledge scores (as percentage out of 100) for control and simulation groups. Asterisk denotes significance when compared to both control and pre-simulation groups at the p <0.05 level

Among students undergoing the simulation intervention, pre-simulation survey results did not vary by semester the clerkship was completed (p=0.94), Ob/Gyn interest (p=0.47), or prior SVD exposure (p=0.22). Simulation students’ survey knowledge scores significantly increased after participating in the simulation (54.5% vs. 76.9%, p< 0.01; Figure 1). Knowledge scores following completion of the Ob/Gyn clerkship were similar to post-simulation scores (76.9% vs. 84.1%, p=0.07; Figure 1).

Knowledge scores post-clerkship were significantly higher for the simulation cohort compared to controls (84.1% vs. 53.3%, p < .01; Figure 1).

### Confidence

Clerkship student confidence significantly increased for all surveyed components of the vaginal delivery immediately following the simulation (p <.01; Table 2).

**Table 2.** Confidence of Intervention Group at Each Stage in Study and Control Group to Perform Each Step of a Vaginal Delivery, Rated on a Likert Scale From 1 (“I Am Not at all Confident” to 4 (I Am Very Confident, Even Independently, With Back-Up for Problems”)

Survey item: In a vaginal delivery…					​
Group (N)	I Am not at all confident (N (%))	I Am somewhat confident, with close hand-on-hand assistance (N (%))	I Am confident, with minimal supervision (N (%))	I Am very confident, even independently, with back-up for problems (N (%))	Mean confidence ± SEM
1. I am comfortable controlling the head.					
Control (54)	29 (53.7%)	14 (25.9%)	10 (18.5%)	1 (1.6%)	1.69 ± 0.12
Pre-Sim (259)	223 (86.1%)	31 (11.9%)	4 (1.5%)	1 (0.3%)	1.16 ± 0.02
Post-Sim (259)	1 (0.35)	49 (18.9%)	136 (52.5%)	73 (28.2%)	3.09 ± 0.04
Post-Clerkship (71)	6 (8.5%)	15 (21.1%)	23 (32.4%)	27 (38.0%)	2.70 ± 0.12
2. I am comfortable delivering the shoulders.					
Control (54)	33 (61.1%)	15 (27.8%)	5 (9.3%)	1 (1.9%)	1.52 ± 0.11
Pre-Sim (259)	228 (88.0%)	27 (10.4%)	2 (0.8%)	2 (0.8%)	1.12 ± 0.02
Post-Sim (259)	3 (1.2%)	62 (23.9%)	139 (53.7%)	55 (21.2%)	2.95 ± 0.04
Post-Clerkship (71)	12 (16.9%)	20 (28.2%)	24 (33.8%)	15 (21.2%)	2.59 ± 0.12
3. I am comfortable with the delivery after the shoulders.					
Control (54)	23 (42.6%)	20 (37.0%)	5 (9.3%)	6 (11.1%)	1.89 ± 0.14
Pre-Sim (259)	211 (81.5%)	36 (13.9%)	9 (3.5%)	3 (0.1%)	1.22 ± 0.023
Post-Sim (259)	7 (0.3%)	40 (15.4%)	136 (52.5%)	75 (29.0%)	3.13 ± 0.04
Post-Clerkship (71)	9 (12.7%)	17 (23.9%)	24 (33.8%)	21 (29.6%)	2.80 ± 0.12
4. I am comfortable delivering the placenta.					
Control (54)	5 (9.3%)	7 (12.9%)	16 (29.6%)	26 (48.1%)	3.17 ± 0.13
Pre-Sim (259)	183 (70.6%)	51 (19.7%)	15 (5.8%)	10 (3.9%)	1.40 ± 0.04
Post-Sim (259)	23 (8.9%)	116 (44.8%)	115 (44.0%)	5 (1.9%)	3.36 ± 0.03
Post-Clerkship (71)	3 (4.2%)	4 (5.6%)	13 (18.3%)	51 (71.8%)	3.58 ± 0.09
5. I am comfortable examining the placenta.					
Control (54)	7 (12.9%)	6 (11.1%)	15 (27.8%)	26 (48.1%)	3.11 ± 0.15
Pre-Sim (259)	175 (67.6%)	58 (22.4%)	14 (5.4%)	12 (4.6%)	1.45 ± 0.78
Post-Sim (259)	38 (14.7%)	113 (43.6%)	101 (39.0%)	8 (3.1%)	3.25 ± 0.04
Post-Clerkship (71)	3 (4.2%)	4 (5.6%)	14 (19.7%)	50 (70.4%)	3.56 ± 0.09

Clerkship student confidence controlling the head, delivering the shoulders, and with delivery following the shoulders was significantly greater immediately following the simulation when compared to following the clerkship (3.1 vs. 2.7, p=0.01; 2.9 vs. 2.6, p=0.02; 3.1 vs. 2.8, p=0.03, respectively). Following the clerkship, students were more confident in delivering and examining the placenta than immediately following the simulation (3.6 vs. 3.4, p=0.03; 3.6 vs. 3.3, p=0.04, respectively).

Post-clerkship student confidence controlling the head, delivering the shoulders, with delivery following the shoulders, delivering the placenta, and examining the placenta was significantly greater than controls (2.7 vs. 1.7, p < .01; 2.6 vs. 1.5, p < .01; 2.8 vs. 1.9, p < .01; 3.6 vs. 3.2, p=0.01; 3.6 vs. 3.1, p < .01, respectively).

Immediately following the simulation, students reported significantly greater confidence in their ability to participate in vaginal deliveries (3.2 vs. 1.6, p < .01) and felt more prepared to attempt a supervised vaginal delivery (1.4 vs. 3.1, p < .01). Following their clerkship, students in the simulation cohort report significantly greater confidence to participate in a vaginal delivery (3.3 vs. 2.5, p <.01) and attempt a supervised delivery (3.2 vs 2.2, p <.01) when compared to students who had previously completed the clerkship without receiving simulation education.

### Experience

**Figure 2.**
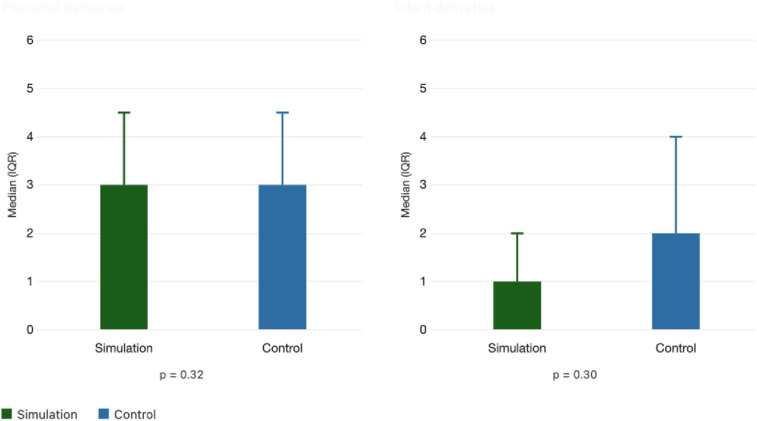
Placental and infant deliveries during the Obstetrics rotation for students who completed an SVD simulation as compared to students who previously completed the clerkship without simulation

Students participated in 3 (IQR 3) placental deliveries and 1 (IQR 2) infant deliveries (Figure 2). Students who completed the simulation at the start of their Obstetrics rotation participated in a similar number of placental deliveries (3 (IQR 3) vs. 3 (IQR 3), p=0.32) and infant deliveries (1 (IQR 2) vs 2 (IQR 4), p=0.3) when compared to students who had previously completed the clerkship without receiving simulation education.

## Discussion

We implemented an SVD lecture and simulation led by near-peer senior medical students using existing teaching and simulation resources. The intervention was associated with improved clerkship students’ knowledge and self-assessed preparedness for their Ob/Gyn rotation. Knowledge, confidence to participate in deliveries, and self-perceived preparedness to perform a vaginal delivery following the clerkship was greater in the simulation cohort than in the control group who had previously completed their clerkship without simulation. Participation in deliveries was not different between the simulation cohort and control group.

Existing studies have shown similar improvements in knowledge scores following SVD lecture and simulation taught by faculty.^10^ No other studies included in our literature review compared post-simulation and post-clerkship knowledge scores for the intervention group. Our study found that knowledge scores increased, though not significantly, following the clerkship. This indicates that the information presented in the SVD lecture and simulation was sustained and may have been reinforced through clinical experiences during the clerkship. However, post-clerkship knowledge was assessed within 1 week of the clerkship (within 4-7 weeks of simulation session), so further research is indicated to determine if knowledge is sustained over a longer amount of time.

Interestingly, all confidence scores related to delivery of the infant decreased from post-simulation to post-clerkship. This may be due to students’ limited experience with infant delivery during their rotation, as students who completed the clerkship participated in a median of 1 infant delivery. The decline in confidence may also reflect their exposure to delivery outside of the controlled simulation environment. In the clinical environment, students likely observed the variability in deliveries, the need for rapid decision-making, and the risk and management of complications, leading them adopt a more realistic, cautious view of their own readiness.

Self-perceived confidence to perform all components of the vaginal delivery improved following the simulation, which is similar to findings in other studies taught by faculty.^1,4,5^ Following the clerkship, students were more confident to participate in delivery and examine the placenta than they were immediately following the simulation. However, there was no significant change in confidence to participate in other components of the vaginal delivery. This may be in part explained by clerkship experience delivering placentas, as students participated in more placenta than infant deliveries.

To our knowledge, this study is the first to demonstrate that Obstetric simulation led by near-peer teachers is effective in improving clerkship students’ knowledge and confidence to participate in SVDs. As the role for simulation in the Obstetric clerkship expands, barriers to simulation, including cost, distribution of teachers and learners, and resources must be addressed.^11^ Our study aimed to address these factors by implementing the lecture and simulation on the first day of the Obstetrics clerkship during protected education time, using a low-fidelity manikin that was previously purchased by our institution for other purposes.

This study is not without limitations. Our control group included students who had completed the Ob/Gyn clerkship in the prior one year without receiving the simulation. The survey was distributed retrospectively to all eligible students and participation was voluntary, introducing potential selection and recall biases. The overall response rate was 28.9%. Students who elected to complete the survey may significantly differ from non-respondents in their interest or knowledge of Ob/Gyn. Additionally, we are unable to determine to what degree the differences found in knowledge and confidence are affected by the length of time since the control student had completed the clerkship. While time since the clerkship may decrease knowledge and confidence for some, others may have gained more exposure and education related to spontaneous vaginal deliveries since their third-year clerkship.

The response rate post-clerkship was notably lower than the pre- and post-simulation surveys. We suspect that this is due to the virtual distribution of the survey following the clerkship. Time was allotted for the pre- and post-simulation surveys prior to and immediately following the in-person lecture and simulation session, which likely increased response rates. The students who responded to the post-simulation survey may have systematically differed than those who did not, introducing selection bias to the post-clerkship data.

Additionally, there are inherent limitations in a non-randomized design. Our novel use of near-peer teachers demonstrated outcomes similar to studies performed on simulation led by faculty, which is important given high demands on clinical faculty. Future studies may consider a randomized control trial comparing near-peer-led to faculty-led SVD simulations on medical student knowledge, confidence and experiences to determine if these outcomes differ per the type of instructor. Additionally, future studies may further investigate students’ preference for near-peer or faculty instructors.

## Conclusion

A near-peer led spontaneous vaginal delivery lecture for undergraduate medical students is a feasible and effective tool for the Ob/Gyn clerkship. Despite limited clinical opportunities to participate in vaginal deliveries during the clerkship, students who participated in near-peer-led simulation demonstrated greater overall knowledge and confidence than historical controls. Near-peer instructors may expand access to meaningful hands-on training while mitigating logistical barriers. Future research should directly compare faculty-led and near-peer-led simulation models, assess long-term knowledge retention, and explore learner preferences to optimize simulation-based learning in the Ob/Gyn clerkship.

## Supplemental Material

Supplemental Material - Near-Peer-Led Spontaneous Vaginal Delivery Simulation Improves Clerkship Students’ Knowledge and Confidence Supplemental Material for Near-Peer-Led Spontaneous Vaginal Delivery Simulation Improves Clerkship Students’ Knowledge and Confidence by Claire M. Schenken, Nisha Kalyanpur, , Malavika Perinchery, Jacqueline L. Moreno, Nicholas Stansbury, Gabriel Medrano-Valle, Sarah M. Page-Ramsey and Erin L. Nelson in Journal of Medical Education and Curricular Development.

## Data Availability

Available upon reasonable request.*
