# Supplementary material for: Near-Peer-Led Spontaneous Vaginal Delivery Simulation Improves Clerkship Students’ Knowledge and Confidence
Source: J Med Educ Curric Dev. 2026 Jun 8;13:23821205261459895. doi: 10.1177/23821205261459895 (PMC13247282; doi:10.1177/23821205261459895)
Supplement: Supplemental Material - Near-Peer-Led Spontaneous Vaginal Delivery Simulation Improves Clerkship Students’ Knowledge and Confidence [file sj-pdf-1-mde-10.1177_23821205261459895.pdf]

Supplemental Digital Content 1. Doc, surveys distributed to participants

Pre-Simulation Survey

1. I identify as:
    a. Female
    b. Male
    c. Non-binary
    d. Prefer to self-describe: ____
    e. Prefer not to say
2. What is your graduation year?
3. When did you begin your Ob/Gyn rotation
4. What best describes your interest in Ob/Gyn?
    a. I plan to go into Ob/Gyn
    b. I am not sure
    c. I do not plan on going into Ob/Gyn
5. What best describes your previous experience with vaginal deliveries?
    a. No previous exposure to obstetrics in a clinical setting
    b. Observed 1 vaginal delivery
    c. Observed 2+ vaginal deliveries
    d. Performed 1 vaginal delivery with assistance
    e. Performed 1 vaginal delivery with minimal supervision or independently
    f. Performed 2+ vaginal deliveries with minimal supervision or independently
    g. Other, please describe: ___________
6. Please rate your current level of confidence by: (1) I am not at all confident; (2) I am somewhat confident, with close hand-on-hand assistance; (3) I am confident, with minimal supervision; (4) I am very confident, even independently, with back-up for problems
    a. I can identify the position and station of the fetus in labor
    b. I can identify the cardinal movements of the fetus during delivery
    c. I am confident I can participate in deliveries
    d. I am ready to attempt a supervised vaginal delivery
7. Please rate your current level of confidence (1-5, as described above): In a vaginal delivery...
    a. I am comfortable controlling the head
    b. I am comfortable delivering the shoulders
    c. I am comfortable with the delivery after the shoulders
    d. I am comfortable delivering the placenta
    e. I am comfortable examining the placenta
8. Stage 4 of labor is the delivery of the placenta
    a. True
    b. False
9. When a patient is 7 cm dilated, they are in stage ______ of labor
    a. I: Latent phase
    b. I: Active phase
    c. II
    d. III

    e. IV
10. Fetal head flexion occurs after crowning
    a. True
    b. False
11. Fill in the blank: There is arrest of labor if there is no cervical change despite ____ hours of adequate contractions
12. The umbilical cord is comprised of:
    a. How many arter(ies)?
        i. 0
        ii. 1
        iii. 2
        iv. 3
        v. 4
    b. How many vein(s)?
        i. 0
        ii. 1
        iii. 2
        iv. 3
        v. 4

Post-Simulation Survey

1. What best describes your interest in Ob/Gyn?
    a. I plan to go into Ob/Gyn
    b. I am not sure
    c. I do not plan on going into Ob/Gyn
2. Please rate your current level of confidence by: (1) I am not at all confident; (2) I am somewhat confident, with close hand-on-hand assistance; (3) I am confident, with minimal supervision; (4) I am very confident, even independently, with back-up for problems
    a. I can identify the position and station of the fetus in labor
    b. I can identify the cardinal movements of the fetus during delivery
    c. I am confident I can participate in deliveries
    d. I am ready to attempt a supervised vaginal delivery
3. Please rate your current level of confidence (1-5, as described above): In a vaginal delivery...
    a. I am comfortable controlling the head
    b. I am comfortable delivering the shoulders
    c. I am comfortable with the delivery after the shoulders
    d. I am comfortable delivering the placenta
    e. I am comfortable examining the placenta
4. Stage 4 of labor is the delivery of the placenta
    a. True
    b. False
5. When a patient is 7 cm dilated, they are in stage ______ of labor
    a. I: Latent phase

b. I: Active phase
c. II
d. III
e. IV
6. Fetal head flexion occurs after crowning
   a. True
   b. False
7. Fill in the blank: There is arrest of labor if there is no cervical change despite ____ hours of adequate contractions
8. The umbilical cord is comprised of:
   a. How many arter(ies)?
      i. 0
      ii. 1
      iii. 2
      iv. 3
      v. 4
   b. How many vein(s)?
      i. 0
      ii. 1
      iii. 2
      iv. 3
      v. 4

Post-Clerkship Survey

1. What best describes your interest in Ob/Gyn?
   a. I plan to go into Ob/Gyn
   b. I am not sure
   c. I do not plan on going into Ob/Gyn
2. How many times did you participate in the delivery of a placenta during your rotation?
3. How many times did you participate in the delivery of a baby during your rotation? (independently with supervision or with a resident i.e. hand-on-hand; do not include deliveries only watched or those that you participated in delivery of placenta but not baby)
4. Please rate your current level of confidence by: (1) I am not at all confident; (2) I am somewhat confident, with close hand-on-hand assistance; (3) I am confident, with minimal supervision; (4) I am very confident, even independently, with back-up for problems
   a. I can identify the position and station of the fetus in labor
   b. I can identify the cardinal movements of the fetus during delivery
   c. I am confident I can participate in deliveries
   d. I am ready to attempt a supervised vaginal delivery
5. Please rate your current level of confidence (1-5, as described above): In a vaginal delivery...
   a. I am comfortable controlling the head
   b. I am comfortable delivering the shoulders

    c. I am comfortable with the delivery after the shoulders
    d. I am comfortable delivering the placenta
    e. I am comfortable examining the placenta
6. Stage 4 of labor is the delivery of the placenta
    a. True
    b. False
7. When a patient is 7 cm dilated, they are in stage _______ of labor
    a. I: Latent phase
    b. I: Active phase
    c. II
    d. III
    e. IV
8. Fetal head flexion occurs after crowning
    a. True
    b. False
9. Fill in the blank: There is arrest of labor if there is no cervical change despite ____ hours of adequate contractions
10. The umbilical cord is comprised of:
    a. How many arter(ies)?
        i. 0
        ii. 1
        iii. 2
        iv. 3
        v. 4
    b. How many vein(s)?
        i. 0
        ii. 1
        iii. 2
        iv. 3
        v. 4

Survey for Students Who Previously Completed the Clerkship Without Simulation

1. I identify as:
    a. Female
    b. Male
    c. Non-binary
    d. Prefer to self-describe: ____
    e. Prefer not to say
2. What is your graduation year?
3. When did you begin your Ob/Gyn rotation

4. What best describes your interest in Ob/Gyn?
    a. I plan to go into Ob/Gyn
    b. I am not sure
    c. I do not plan on going into Ob/Gyn
5. How many times did you participate in the delivery of a placenta during your rotation?
6. How many times did you participate in the delivery of a baby during your rotation?
7. Please rate your current level of confidence by: (1) I am not at all confident; (2) I am somewhat confident, with close hand-on-hand assistance; (3) I am confident, with minimal supervision; (4) I am very confident, even independently, with back-up for problems
    a. I can identify the position and station of the fetus in labor
    b. I can identify the cardinal movements of the fetus during delivery
    c. I am confident I can participate in deliveries
    d. I am ready to attempt a supervised vaginal delivery
8. Please rate your current level of confidence (1-5, as described above): In a vaginal delivery...
    a. I am comfortable controlling the head
    b. I am comfortable delivering the shoulders
    c. I am comfortable with the delivery after the shoulders
    d. I am comfortable delivering the placenta
    e. I am comfortable examining the placenta
9. Stage 4 of labor is the delivery of the placenta
    a. True
    b. False
10. When a patient is 7 cm dilated, they are in stage ______ of labor
    a. I: Latent phase
    b. I: Active phase
    c. II
    d. III
    e. IV
11. Fetal head flexion occurs after crowning
    a. True
    b. False
12. Fill in the blank: There is arrest of labor if there is no cervical change despite ____ hours of adequate contractions
13. The umbilical cord is comprised of:
    a. How many arter(ies)?
        i. 0
        ii. 1
        iii. 2
        iv. 3
        v. 4
    b. How many vein(s)?
        i. 0

ii. 1
iii. 2
iv. 3
v. 4
